# How public discourse on medical AI shapes governance expectations: a Weibo-based mixed-methods study from China

**DOI:** 10.3389/fpubh.2025.1693397

**Published:** 2025-11-21

**Authors:** Ting Jiang, Na Wei, Qiang Yan, Weicheng Ye

**Affiliations:** 1 School of Economics and Management, Beijing University of Posts and Telecommunications, Beijing, China; 2 School of Economics and Management, Tsinghua University, Beijing, China

**Keywords:** medical artificial intelligence, public perception, social media analysis, cognition-affect-behavior model, ethical governance, policy advocacy

## Abstract

**Objective:**

Public perceptions of medical artificial intelligence (AI) directly influence its implementation and governance. While most existing research focuses on Western contexts, there is limited exploration of public responses in collectivist cultures and state-driven healthcare systems like China, particularly regarding the dynamic interplay of cognition, affect, and behavior. This study aims to fill this gap by examining public discourse on medical AI in China, with a specific focus on topic landscape, sentiment distribution, and the Cognition-Affect-Behavior (CAB) mechanisms driving governance.

**Methods:**

We collected 12,356 valid Weibo posts on medical AI from January 2022 to December 2024. The Latent Dirichlet Allocation (LDA) topic modeling identified key topics, sentiment analysis assessed emotional tendencies, and grounded theory analysis was applied to 1,000 posts using open, axial, and selective coding to construct a theoretical model.

**Results:**

The findings revealed that public discussions covered eight key topics, categorized into three dimensions: foundational drivers of medical AI development, application domains of medical AI, and societal benefits and risks challenges. All topics exhibited a coexistence of positive and negative emotions. The CAB model showed that, cognitively, the public emphasized the human core of healthcare, while acknowledging AI’s efficacy, leading to a collaborative augmentation model for the physician-AI integration, where decision-making is physician-led, and AI serves as a supportive tool. Emotionally, the public expressed both amazement at AI’s capabilities and expectations for physician-AI integration, alongside resistance to AI and anxiety about the physician-AI integration. Behaviorally, three proactive agency governance strategies were observed, which either reinforced or recalibrated existing cognitive frameworks.

**Conclusion:**

This study provides valuable insights into the public’s cognitive and emotional responses, as well as proactive behaviors toward medical AI in China. It also highlights the emergence of bottom-up accountability mechanisms, where civic engagement shapes the development of AI governance frameworks in healthcare.

## 1 Introduction

### 1.1 Background of medical AI and the critical role of public perspectives

The rapid advancement and widespread implementation of medical artificial intelligence (AI) have become a central focus in the global healthcare technology field, academic research, and policy development (1, 2). Medical AI encompasses a diverse range of technologies, including computer vision systems for imaging interpretation (3), predictive algorithms for clinical resource management (4) and more recently, natural language processing tools such as large language models (LLMs) (5, 6). These technologies are being applied across various domains—such as diagnostic imaging, clinical decision support, patient triage, and postoperative monitoring—where they significantly enhance efficiency, accuracy, and personalization in healthcare delivery (7, 8), ultimately improving patient outcomes (9, 10). However, these benefits coexist with profound ethical tensions in public health contexts—including algorithmic opacity, data privacy risks, and accountability gaps—that fuel public ambivalence and directly challenge the responsible deployment of AI technologies (11, 12). Consequently, the widespread adoption of medical AI is increasingly constrained by public acceptance of its societal and ethical implications rather than technical feasibility (11, 12).

The public plays a critical role in the implementation of medical AI, extending far beyond merely being recipients of healthcare (13). They occupy three key roles: (1) as ultimate beneficiaries of medical services, whose cognitive biases and attitudes influence their willingness to embrace and promote technology (14); (2) as indirect participants in policy development, whose opinions help guide the development and implementation of healthcare technologies (11); and (3) as sensors of societal ethical boundaries, whose perceptions of acceptable trade-offs, such as data privacy, algorithmic fairness, system reliability, and accountability mechanisms, signal the limits of public tolerance for AI deployment (15). However, these tensions, manifested as public biases, emotional responses, and behavioral resistance, hinder medical AI implementation while eroding trust in healthcare systems (16). When such sentiment manifests as skepticism or fear, it creates tangible barriers to clinical integration (17). Therefore, decoding these multidimensional concerns becomes essential for realizing a ‘human-centered’ digital health future, where technology aligns with societal ethical imperatives.

### 1.2 Literature review and research gap

Many studies have explored public discussions surrounding medical AI, primarily utilizing survey-based and structured interview methods. For instance, surveys frequently employ instruments such as the Likert 5-point scale to assess public attitudes (18), perceived reliability, and willingness to adopt medical AI (11), yielding reliable and quantifiable data. Similarly, several studies have utilized interviews to investigate public perceptions of the demand for medical AI (19), as well as public views on the potential benefits and emerging ethical concerns associated with medical AI (15) and opinions regarding the use of health data in AI research (20). However, these methods are often small-scale and rely on predefined questions, which limits their ability to offer in-depth, nuanced insights into the complexity of public perceptions.

As an alternative, some studies have turned to social media data to capture public sentiment. For example, Gao et al. (21) analyzed Weibo posts to explore broad topics of public discussion and attitudes toward medical AI. They identified three primary categories of discussion: technology and application, industry development, and societal impact (21). Their findings revealed that public sentiment toward medical AI was predominantly positive (21). Nevertheless, both methodological streams struggle to capture the tripartite interaction among: (a) cognitive assessments (e.g., opacity of medical AI decisions), (b) affective responses to risks (e.g., distrust in algorithmic fairness), and (c) consequent behavioral demands for ethical governance (e.g., policy reform advocating transparency)—despite separately documenting some dyadic relationships (21–24).

Moreover, much of the existing research on medical AI predominantly originates from Western contexts (17, 25), failing to adequately explore how collectivist cultural paradigms shape ethical considerations in the adoption of digital health technologies. This research gap is especially pronounced in China, where state-led policies and nationally endorsed technological developments fundamentally shape public engagement with medical AI (26). For instance, China’s healthcare policies, which prioritize universal health coverage and social equity, cultivate a distinct public propensity to trust and accept state-endorsed healthcare AI systems (26). These systemic differences highlight the urgent need to broaden medical AI research beyond Western epistemological frameworks, with a particular focus on China’s unique socio-political construction of AI ethics through culturally embedded pathways.

### 1.3 Research objectives

This study integrates three complementary analytical methods: Latent Dirichlet Allocation (LDA) topic modeling, sentiment analysis, and grounded theory, to analyze a dataset of 12,356 social media posts from the Chinese public. First, topic modeling identifies key issues within public discourse; next, sentiment analysis quantifies sentiment polarity and intensity; and finally, grounded theory refines the relationship between cognition, emotion, and behavior, ultimately developing a Cognition–Affect–Behavior (CAB) framework. Guided by the CAB framework, the study explores several critical questions: What specific aspects of medical AI concern the Chinese public, shaped by the country’s unique socio-political landscape? What emotional responses do these concerns trigger? How do cognitive factors shape or amplify these emotional responses, leading to emotional conflict? And how do these tensions translate into behavioral demands, such as calls for education or regulatory reform?

## 2 Methodology

### 2.1 Data collection and processing

As one of China’s leading platforms for public discourse, Sina Weibo provides a rich data source for capturing societal perspectives in an authentic and dynamic manner (27). Given this, the application of LDA topic modeling, sentiment analysis, and grounded theory to analyze unstructured Weibo data offers a robust and systematic approach for examining public concerns, sentiment distribution.

To comprehensively capture public discussions on medical AI, this study collected Weibo posts from discussion threads under hashtags such as #*WillAIDoctorsBePossible* (#A*I 医生会成为可能吗*), #*WouldYouAcceptAIDoctors* (*#你接受AI医生给你看病吗*), and #*WillAIDoctorsReplaceHumanDoctors* (#*医生会被AI取代吗*) as keyword filters. Data collection was conducted using Python’s requests library, leveraging browser login cookies to access Weibo’s dynamic web pages. Upon receiving webpage responses, BeautifulSoup and lxml were employed for webpage parsing, with XPath techniques used for precise extraction of Weibo posts containing relevant keywords. Posts were collected from January 2022 to December 2024, enabling an extensive examination of public discussions on medical AI.

In the data preprocessing stage, several steps were undertaken to clean and refine the raw dataset. First, Jieba, a widely used Python library for Chinese word segmentation, was applied to tokenize the text. Second, a customized stopword list was developed by integrating general stopword corpora (e.g., the Baidu Stopword List) with domain-specific terms relevant to medical AI. Additionally, noise filtering was performed to eliminate duplicate content, advertisements, and excessively short texts. Following these preprocessing steps, a total of 12,356 valid Weibo posts were retained, forming the foundational dataset for subsequent analysis.

### 2.2 LDA topic analysis

To systematically uncover the key topics in public dicsussion on medical AI, this study employed LDA topic modeling. This approach enables the identification of latent thematic structures within large-scale textual data, facilitating a comprehensive analysis of discussion scope and viewpoint diversity (28).

The first step in the LDA modeling process involved determining the optimal number of topics by evaluating perplexity and topic coherence scores (28, 29). Perplexity measures how well the model generalizes to unseen data, while coherence scores assess the semantic consistency of words within each topic. A lower perplexity value suggests better generalizability, whereas a higher coherence score indicates stronger internal consistency (29).

### 2.3 Sentiment analysis

To compare public sentiment across different topics, we adopted sentiment analysis using the SnowNLP library to evaluate the sentiment orientation of each LDA-identified topic. Specifically, sentiment polarity scores are computed for each post using SnowNLP, and the corresponding sentiment intensity is aggregated at the topic level (30). To ensure a more accurate representation of sentiment trends, topic-wise sentiment scores are weighted based on the probability distribution of topics within each post, yielding an overall sentiment intensity measure for each topic.

### 2.4 Grounded theory analysis

Although LDA topic modeling and sentiment analysis effectively capture surface-level discourse patterns through lexical co-occurrence, they are limited in their ability to uncover deeper semantic structures and the affective mechanisms that drive public engagement (31). Specifically, these methods do not adequately address how cognitive factors shape and amplify emotional responses, ultimately leading to emotional conflict (31). To bridge this gap, we employed grounded theory as a methodological framework, utilizing manual coding and inductive abstraction to systematically extract underlying conceptual patterns from the dataset (32). This approach allows for a deeper exploration of the cognitive, affective, and behavioral dimensions of public engagement with medical AI, offering richer insights into the emotional and behavioral dynamics involved.

Furthermore, to deepen our analysis, we applied the CAB model as a heuristic lens, rather than a rigid analytical framework (33). The CAB model conceptualizes public engagement through three interconnected elements: cognitive orientations, affective tendencies, and behavioral strategies. This flexible framework allows us to interpret discussions on the benefits and risks of medical AI with greater depth, considering not only the content of the discourse but also the underlying affective and cognitive drivers.

For data selection, we randomly extracted 1,000 posts from the LDA-derived corpus. We then conducted an iterative coding process using NVivo 12, following the standard phases of open coding, axial coding, and selective coding (34). The coding process for this study was conducted independently by two researchers, each with expertise in public health and social science. Before beginning the formal coding, both researchers participated in structured training sessions. These sessions were designed to ensure a thorough understanding of the research objectives, familiarize the coders with the initial coding scheme, and establish a consensus on key concepts, thereby providing a consistent foundation for the independent coding that followed.

To establish intercoder reliability, the two researchers independently performed open coding on a randomly selected subsample of 100 posts. Upon completing individual coding, we compared results line by line. We then discussed any discrepancies, omissions, and the accuracy of the code labels. This process led to the development of a unified coding standard. Table 1 presents examples of discrepancies, the discussion process, and the final resolutions. All subsequent open coding was carried out in accordance with this agreed-upon standard, with additional discussions held whenever uncertainties arose. This iterative process not only enhanced intercoder reliability but also ensured that the code definitions were precise and that the analytical insights were firmly grounded in the raw data.

**Table 1:** Examples of the initial coding phase.

Original post (paraphrased for anonymity)	Coder A’s initial code	Coder B’s initial code	Discrepancy analysis	Outcome
A post emphasizing that while modern technology is advanced, matters of life and death in healthcare demand the utmost importance and allow no room for error.	No code applied	Ultra-low error tolerance in the healthcare industry	The discrepancy arose due to differences in the depth of conceptual extraction, resulting in an omission by Coder A.	Consensus Code: Perception of ultra-low error tolerance in the industry
A user comment noting that AI can sometimes produce incorrect or nonsensical information subtly, requiring humans to have the ability to discern its accuracy.	AI’s contextual reliability constraints	Information discernment competence	The discrepancy stems from differing analytical dimensions: Coder A identified an AI attribute, while Coder B focused on a public competency.	Consensus Code: AI’s Contextual reliability constraints & information discernment competence
An observation that when interacting with patients, there is often a need for emotional care and support, which is seen as a limitation of AI.	AI-Limited affective engagement	Humanistic empathy	Coder B identified humanistic empathy, while Coder A considered it merely as descriptive context, thus not requiring a separate code.	Consensus Outcome: code humanistic empathy not adopted.

In the subsequent phases of axial and selective coding, We collaborated to refine and synthesize the concepts identified during open coding. Through continuous discussion, we grouped related concepts, constructed core categories, and developed a comprehensive theoretical framework that integrated the full dataset. To ensure the robustness and validity of our findings, an additional 200 posts were re-coded until theoretical saturation was reached, meaning no new concepts emerged, thus confirming the comprehensiveness of the coding process.

## 3 Results

### 3.1 Key public topics on medical AI

**Figure 1:**
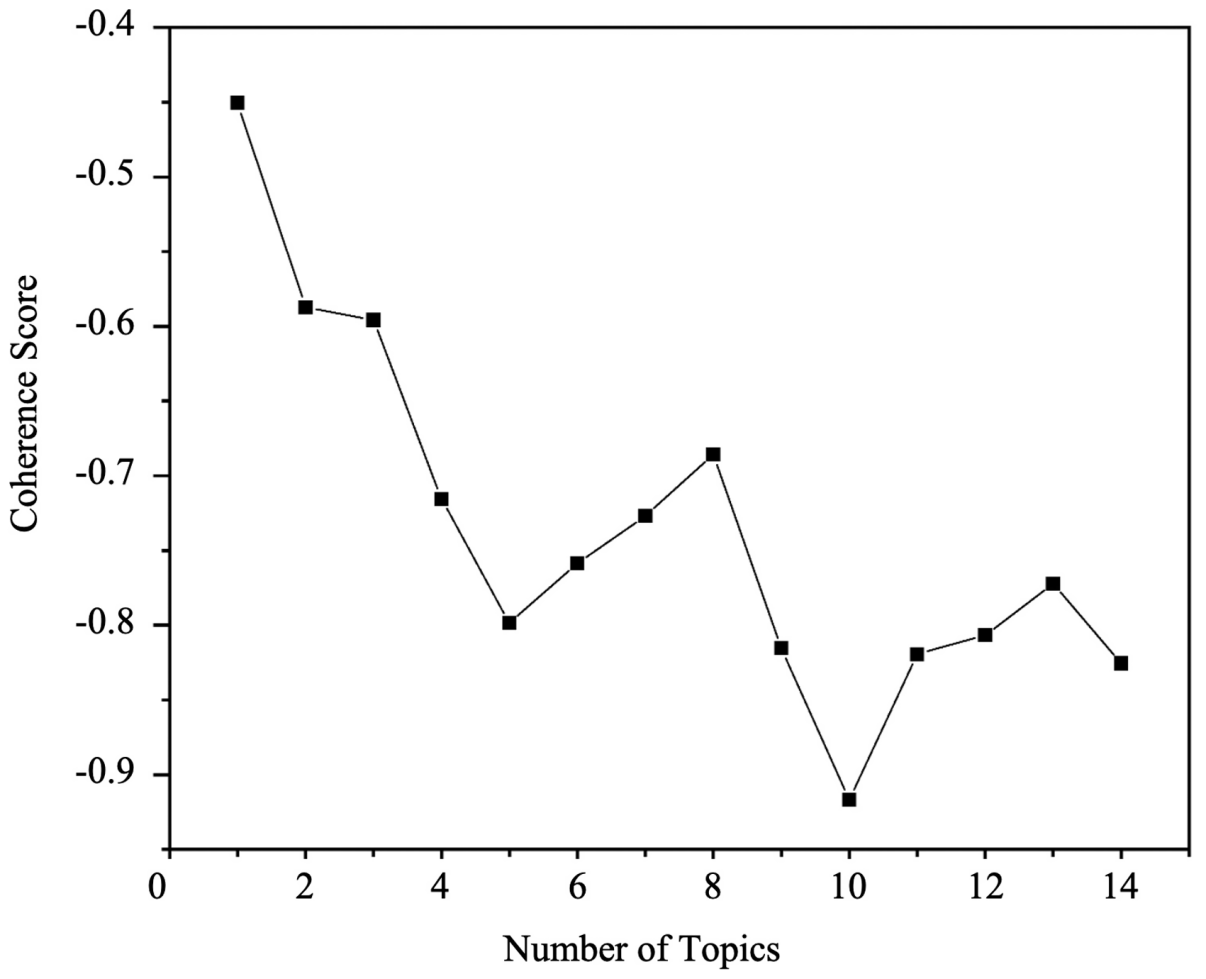
Identification of the optimal topic number (K = 8) for LDA modeling. The coherence score peaks at K = 8, indicating the optimal balance between topic distinctiveness and interpretability. This evidence-based selection ensures that the model captures a comprehensive range of themes while minimizing the risk of overfitting.

The perplexity analysis revealed that when the number of topics exceeded 13, the model exhibited signs of overfitting. Using the elbow method (29), the initial estimate suggested that the optimal number of topics lay within the range of 5 to 13. To accurately identify the point of peak interpretability within this range, we calculated the coherence score for each candidate value of K. As shown in Figure 1, the coherence curve reveals a clear peak at K = 8, indicating the optimal balance between topic distinctiveness and semantic clarity, beyond which the score begins to plateau. This peak suggests that a model with 8 topics strikes the ideal balance between broad coverage and focused, interpretable themes. Based on the convergence of both perplexity and coherence evidence, we selected K = 8 for the final LDA model, ensuring robust performance while minimizing the risk of overfitting.

Building on the clustering results from the LDA model and the distribution of high-frequency keywords (see Table 2), we systematically identified, labeled, and categorized the key topics, which can be broadly classified into three interrelated dimensions: the foundational drivers of medical AI development, its primary application domains, and the societal implications and risks of medical AI. These dimensions provide a comprehensive framework for understanding the various facets of medical AI discussed in the dataset. The following subsections provide a detailed analysis of each dimension.

**Table 2:** Topic distribution of public posts on medical AI.

Identified topics	High-frequency keywords
Topic 1: Digital and intelligent transformation of the healthcare industry	Healthcare, intelligence, health, smart, technology, data, Baidu, product, platform, Internet
Topic 2: Technological innovations in medical AI	Model, research, drug, development, data, algorithm, training, generation, science, prediction
Topic 3: National strategies and global collaborations	China, the USA, global, world, cooperation, culture, consumption, development, growth, policy
Topic 4: Development of the digital economy and innovation ecosystem	Industry, entrepreneurship, digital, economy, technology, system, center, institution, city, service
Topic 5: Market dynamics and investment trends	Market, technology, investment, index, sector, healthcare, stocks, brain-computer interface, growth, capital
Topic 6: Applications of AI in assisted diagnosis and treatment	Doctor, diagnosis, assistance, patient, clinical, improvement, accuracy, plan, treatment, tumor
Topic 7: AI-driven biomedical research and innovations	Gene, testing, disease, analysis, university, research, healthcare, the Lancet, publication
Topic 8: Societal benefits and risk challenges	Society, capability, work, education, learning, impact, privacy, risk, ethics, challenges

#### 3.1.1 Foundational drivers of medical AI development

The first set of topics pertains to the fundamental drivers shaping the development of medical AI. Topic 1 emerges from discussions centered on keywords such as “intelligent,” “data,” “product,” “Internet,” and “Baidu,” reflecting the accelerating digital transformation of the healthcare sector. Driven largely by major technology firms, this transformation extends beyond technological advancements to a fundamental restructuring of healthcare service models. Based on this pattern, we conceptualize this topic as “digital and intelligent transformation of the healthcare industry.”

Topic 2 centers on discussions featuring keywords such as “model,” “data,” “algorithm,” and “generation,” highlighting the foundational role of large-scale models and big data analytics in the evolution of medical AI. As critical enablers of AI-driven healthcare applications, these advancements are reshaping medical decision-making by introducing data-driven precision and automation. Reflecting this technological trajectory, we define this topic as “technological innovations in medical AI.”

Topic 3 is anchored in discussions featuring keywords such as “China,” “policy,” “global,” “world,” and “cooperation” emphasizing the critical role of national strategic planning and policy frameworks in shaping the trajectory of medical AI. This top-down approach not only fosters domestic innovation but also underscores the necessity of global cooperation in AI governance and development. Based on this insight, we define this topic as “national strategies and global collaborations.”

Topic 4 is characterized by keywords such as “digital,” “economy,” and “system,” reflecting discussions on the formation of a digital economic ecosystem that supports medical AI development. This ecosystem not only establishes institutional frameworks and operational infrastructures but also serves as a key driver of value creation in healthcare services. Accordingly, we designate this topic as “development of the digital economy and innovation ecosystem.”

Topic 5 revolves around keywords such as “market,” “technology,” “investment,” and “capital,” highlighting the intricate interplay among market mechanisms, technological progress, and financial investments. This dynamic relationship fuels the expansion of the medical AI sector while optimizing the strategic allocation of innovation resources. Given these insights, we conceptualize this topic as “market dynamics and investment trends.”

Collectively, these five topics constitute the foundational pillars of medical AI development, encompassing national strategies, policy directives, technological innovation, resource infrastructure, the digital economy, and market-driven investments. The interwoven nature of these factors not only drives the digital and intelligent transformation of the healthcare industry but also enhances systemic efficiency, optimizes resource distribution, and ultimately fosters the modernization of healthcare systems.

#### 3.1.2 Application domains of medical AI

Beyond its foundational aspects, medical AI is distinguished by its diverse application domains, particularly in clinical decision support and biomedical research. These two areas exemplify how AI-driven technologies are reshaping both healthcare delivery and scientific discovery.

Topic 6, identified by keywords such as “assistance,” “diagnosis,” “accuracy,” and “improvement,” highlights the pivotal role of medical AI in enhancing diagnostic precision and optimizing clinical workflows. By improving the accuracy of medical assessments and streamlining service efficiency, AI not only elevates the quality of healthcare but also drives fundamental transformations in healthcare delivery models. Given these advancements, we define this topic as “applications of AI in assisted diagnosis and treatment.”

Topic 7, characterized by keywords such as “gene,” “disease,” “university,” “research,” and “the Lancet,” underscores the integration of AI technologies into biomedical research. This shift marks a departure from traditional evidence-based methodologies toward data-driven, AI-powered scientific discovery. Through interdisciplinary collaboration, AI has accelerated breakthroughs in genomics, disease prediction, and personalized medicine, reinforcing its transformative impact on public health and medical knowledge production. Reflecting this evolution, we classify this topic as “AI-driven biomedical research and innovations.”

Notably, while these two topics primarily encapsulate technological applications and scientific advancements, they do not exist in isolation. Rather, they are embedded within a broader socio-technical ecosystem, shaped by regulatory frameworks, strategic resource allocation, capital investment trajectories, and the continuous evolution of AI technologies. This interconnected landscape underscores the reciprocal interplay between medical AI and the structural, economic, and institutional forces that mediate its development and integration into society.

#### 3.1.3 Societal benefits and risk challenges of medical AI

Public discussions on medical AI extend beyond technological advancements and applications to its broader societal implications and governance challenges. Topic 8, identified by keywords such as “society,” “capability,” “work,” “education,” “privacy,” “ethics,” and “challenges,” not only acknowledges AI’s capabilities but also captures prevalent concerns related to shifts in occupational structures, changes in education systems, and critical issues of data privacy and ethics. Accordingly, we classify this topic as “societal benefits and risk challenges.”

The high-frequency keywords reveal that public concerns primarily focus on two major challenges arising from medical AI implementation. First, the impact on healthcare workforce structures and medical education systems is widely discussed. There is clear evidence that medical AI is altering traditional employment patterns in healthcare, particularly for diagnostic specialists. This transformation necessitates significant adjustments in workforce planning and medical training programs.

Second, ethical and governance problems generate sustained public attention. Three issues dominate these debates: (1) how patient data privacy is protected when AI systems process medical records, (2) whether AI diagnostic tools demonstrate consistent accuracy and fairness across diverse patient groups, and (3) who should be held responsible when AI-assisted decisions lead to medical errors. These concerns highlight crucial gaps in current regulations overseeing medical AI applications.

While acknowledging AI’s potential to improve healthcare efficiency and access, these findings underscore serious challenges that demand immediate attention from policymakers and healthcare administrators.

### 3.2 Distribution of public sentiments on medical AI

**Figure 2:**
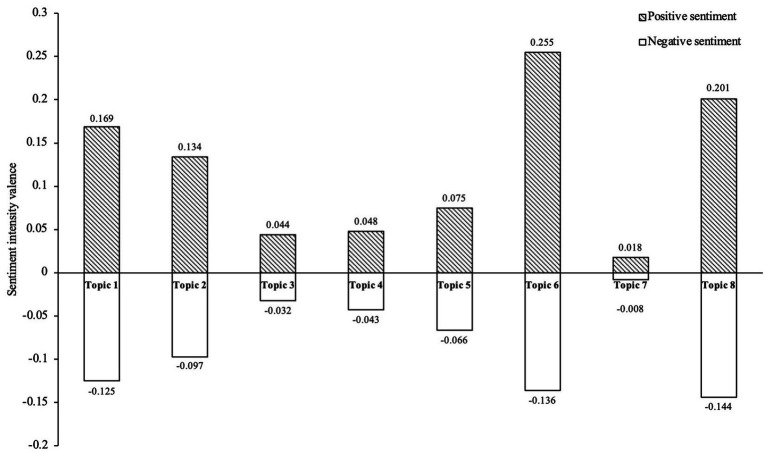
Topic-dependent and dual-edged public sentiment toward medical AI. Analysis reveals that public sentiment toward medical AI is both polarized and topic-dependent. The most intense and polarized sentiments are observed in relation to clinical and societal topics (e.g., Topics 6 & 8), whereas policy-oriented topics (e.g., Topics 3, 4, 7) elicit more neutral responses, reflecting how public engagement is influenced by the perceived relevance of the topic to personal healthcare concerns.

Sentiment analysis reveals a consistent pattern of public sentiments across all eight topics, with each eliciting both positive and negative responses. This duality is captured in Figure 2, which plots sentiment intensity scores (*Y*-axis) against the topics (*X*-axis). Here, positive values indicate a predominance of positive sentiment, while negative values reflect prevailing negative concerns, quantifying the strength of public feeling toward each topic. This visualization underscores that public perception of medical AI is not monolithic but fundamentally dual-edged, marked by a simultaneous recognition of its benefits and risks. The following analysis examines these sentiment distributions in detail.

Among all identified topics, Topic 6 “applications of AI in assisted diagnosis and treatment” stands out in Figure 2 as exhibiting the highest sentiment intensity, with a markedly positive score of 0.255 alongside a notable negative score of −0.136. This pronounced polarity reflects strong public recognition of AI’s potential to enhance diagnostic accuracy, improve clinical efficiency, and expand healthcare access, which is tempered by significant concerns over technological uncertainties such as safety, data privacy, algorithmic bias, and misdiagnosis risks. Overall, this dual sentiment suggests that while the public recognizes the benefits of AI-driven healthcare, they also express significant concerns about its potential risks.

A similar pattern of high emotional engagement is observed in Topic 8 “societal benefits and risk challenges,” which also shows pronounced sentiment intensity in Figure 2, with a positive score of 0.201 and negative score of −0.144. Public optimism in this topic stems from the perception of AI as an assistive tool, operating within fixed logical frameworks that complement rather than replace medical professionals. This view reinforces public confidence in the essential role of healthcare providers. In contrast, negative sentiment is driven by concerns about the long-term societal implications of AI, including potential transformations in the workforce, shifts in medical education, and changes in professional responsibilities.

Other topics exhibiting high sentiment intensity include Topic 1, Topic 2, and Topic 5. The robust positive sentiment associated with these topics reflects public approval and high expectations for AI-driven industrial transformation, technological advancements, and market expansion, which are widely seen as key drivers of smart healthcare, efficiency improvements, and resource optimization. However, the concurrent negative scores reveal persistent public concerns regarding technological limitations, insufficient data privacy protections, market uncertainties, and ongoing ethical and regulatory challenges.

In contrast, as visualized in Figure 2, Topic 7 “AI-Driven Biomedical Research and Innovations,” Topic 3 “National Strategies and Global Collaborations,” and Topic 4 “Development of the Digital Economy and Innovation Ecosystem” exhibit relatively balanced sentiment distributions, with positive sentiment scores not exceeding 0.05 and negative sentiment scores not falling below −0.05. This near-neutral sentiment profile suggests these topics elicit more measured public responses, likely because their specialized and policy-oriented nature makes them less directly relevant to immediate public concerns. Furthermore, these domains generally benefit from broader social consensus and higher cognitive barriers, which fosters more rational and less emotionally charged public perceptions.

Overall, public sentiment toward medical AI exhibits a distinct duality—enthusiastic anticipation of technological advancements coexisting with a cautious awareness of potential risks and uncertainties. This pattern reflects not only perceptions of AI’s capabilities and future prospects but also more nuanced assessments of its integration into society.

### 3.3 Cognition-affect-behavior model of public engagement with medical AI

Building on the sentiment analysis results and the findings from the LDA topic modeling, we conducted a grounded theory analysis on posts related to *Topic 8: Societal Benefits and Risk Challenges*. This theme was selected because it encapsulates the fundamental governance dilemma: balancing technological promise against societal risks. The observed emotional conflicts reflect deeper ethical negotiations about healthcare’s humanistic foundations, making Topic 8 essential for understanding how cognition shapes societal responses to emerging technologies.

Using NVivo 12 software, we conducted open coding, identifying 45 initial categories that captured key dimensions such as the multifaceted complexity of the healthcare industry, satisfaction with medical AI technologies, and the balanced openness and caution toward AI adoption. These categories were further refined into nine subcategories through axial coding, focusing on aspects like the healthcare industry-specific attributes, physician core competencies, and medical AI characteristics. In the selective coding phase, these subcategories were integrated within the CAB framework, organizing them into three core dimensions: cognition, affect, and behavioral dimension (see Table 3).

**Table 3:** Multi-level coding results.

Core category	Subcategory	Initial category
Cognitive dimension	Healthcare industry-specific attributes	Multifaceted complexity
		Ultra-low error tolerance
		Humanistic-ethical imperatives
	Physician core competencies	Clinical experience integration
		Humanistic empathy and compassion
		Medical expertise and clinical judgment
		Diagnostic adaptability
		Patient-centered communication proficiency
	Medical AI characteristics	High diagnostic efficiency
		Limited affective engagement
		Contextual reliability constraints
		Limited human-like cognitive processing
		Decision opacity
	Physician-AI integration	Collaborative augmentation model
		Task-specific substitution model
Affective dimension	Attitudes toward AI technology	Amazement
		Appreciation
		Satisfaction
		Gratitude
		Pride
		Anticipation
		Confidence
		Concern
		Resistance
		Skepticism
	Attitudes toward physician-AI integration	Appreciation
		Satisfaction
		Optimism
		Reassurance
		Anticipation
		Resignation
		Concern
		Fear
		Anxiety
Behavioral dimension	Cultivating technological rationality and cautious awareness	Balanced openness and caution
		Comprehensive risk–benefit assessment awareness
		Leadership in collaborative decision-making
		Critical thinking against misguidance
	Enhancing information and AI literacy	Information discernment competence
		Foundational technical comprehension
		Critical application and continuous learning
		Independent and innovative thinking
	Advocating policy implementation	Decision reliability and transparency
		Robust data security protocols
		Accountability and rights protection mechanisms

**Figure 3:**
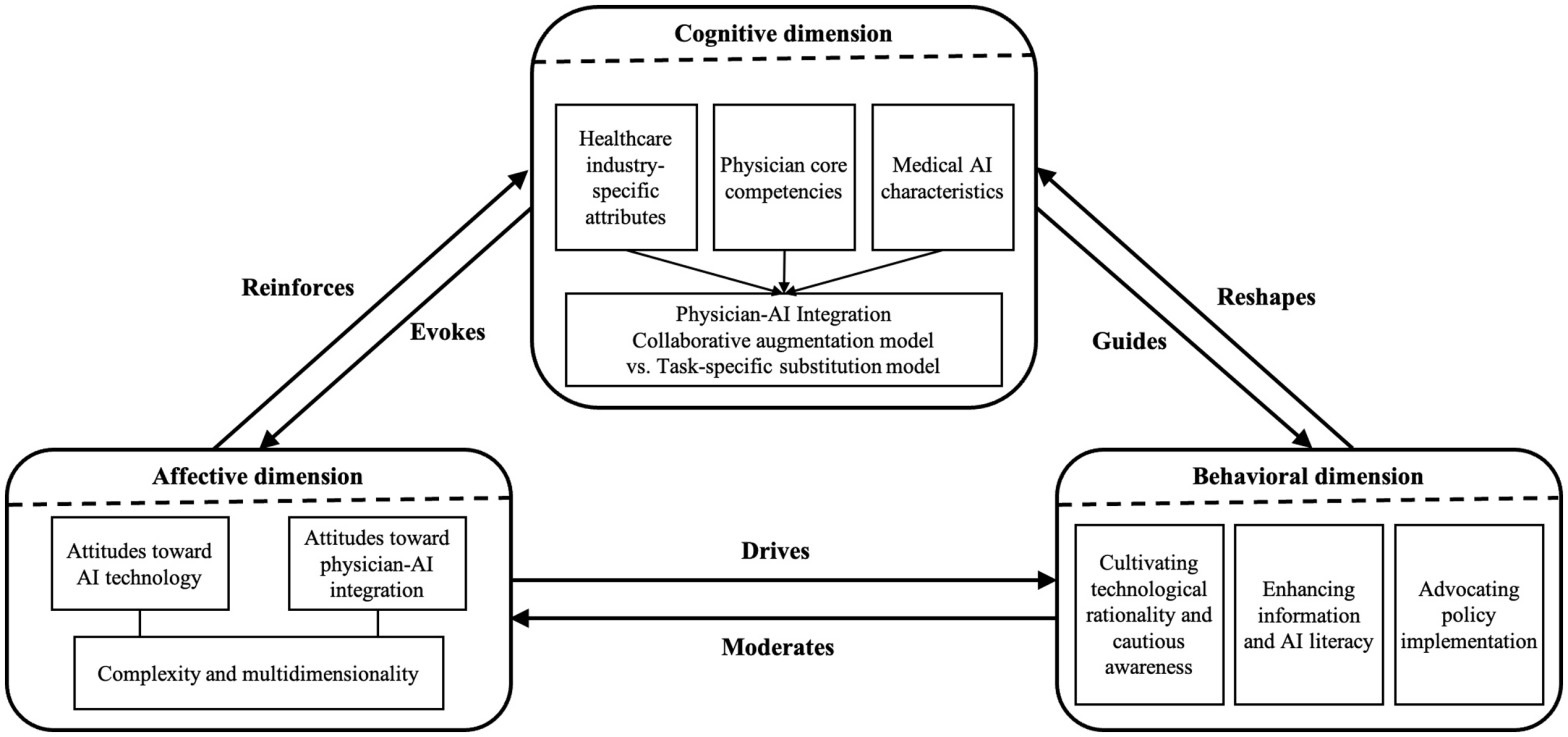
Public engagement with medical AI: A dynamic feedback loop across cognitive, affective, and behavioral dimensions. This framework synthesizes our findings into a cyclical process, demonstrating how public cognition elicits emotional responses that drive specific coping behaviors. The model emphasizes the continuous feedback loop, where these behaviors, in turn, reshape cognitive frameworks, facilitating ongoing societal adaptation and the evolution of public understanding of medical AI.

Our findings on public cognition, affective responses, and behavioral strategies are synthesized into the dynamic, cyclical model presented in Figure 3. This model illustrates the continuous feedback loop through which the public perceives, emotions, and adapts to medical AI. The process begins with cognitive foundations, which trigger emotional reactions; these emotions, in turn, motivate coping behaviors, the outcomes of which ultimately reshape the initial cognitive frameworks, facilitating ongoing societal adaptation.

To ensure theoretical rigor, an additional 200 posts were re-coded after the selective coding phase. No new categories or concepts emerged, confirming that theoretical saturation was achieved. The following sections present detailed analyses based on findings summarized in Table 3 and Figure 3.

#### 3.3.1 Cognitive dimensions

The cycle, as modeled in Figure 3, is initiated by the foundational dimension of public cognition structured around several core perceptions (see Table 3). This perception first acknowledges that healthcare’s complexity extends beyond the diversity of medical conditions and the interdisciplinary nature of medical knowledge. The public further public views healthcare as a domain defined by its ultra-low error tolerance and, crucially, by its humanistic-ethical imperatives, including complex interpersonal relationships, moral obligations, and the need for psychological support. This leads to the expectation that physicians possess not only exceptional professional knowledge and clinical adaptability but also strong communication skills and a high degree of empathy. These rigorous expectations for physician competencies form a critical benchmark against which medical AI is evaluated, shaping the initial input into the cyclical engagement process.

When assessed against this benchmark, medical AI is viewed through a lens of technological duality, a core cognitive assessment captured in our model. It is widely acknowledged for its high diagnostic efficiency and operational efficacy. However, these strengths are tempered by persistent concerns about its contextual reliability constraints, decision opacity, and limited human-like cognitive processing. More fundamentally, as a purely technological entity, medical AI is perceived as inherently lacking essential human attributes such as independent reasoning, emotional intelligence, and empathetic interaction. This perceived limitation is evident in public discourse, where AI is often viewed merely as a technological tool, lacking essential human attributes such as genuine intelligence and autonomous decision-making. This perspective reinforces a prevailing consensus that defines clear boundaries for the role of AI.

Building upon these cognitive frameworks, specifically, the perceptions of healthcare distinctiveness, physician competencies, and AI characteristics outlined in Figure 3, the predominant public opinion advocates for a collaborative augmentation model over a substitutive relationship. This constitutes the fourth cognitive dimension of the model, physician-AI Integration. In this model, medical AI serves as a supportive tool that enhances physician capabilities, who maintain leadership by leveraging their clinical experience and patient-centered communication, while AI provides complementary support through advanced data analytics.

Nevertheless, a subset of the public maintains a more open-minded outlook, expressing optimism that the potential of future technological advancements is limitless. This perspective, informed by AI’s successful integration into specialized tasks, foresees a more dominant role for AI, indicating a spectrum of public readiness that feeds into the ongoing cycle of appraisal and adaptation central to Figure 3.

#### 3.3.2 Affective responses

As illustrated in the model of public engagement, foundational cognitive assessments serve as the initial triggers for complex, multidimensional affective responses. These emotional reactions are not merely outcomes; rather, they act as a pivotal catalyst, facilitating the transition from cognitive perceptions to potential actions within the model.

The public’s emotional response to medical AI technology is defined by a fundamental technological paradox, which is reflected in the dual sentiment observed in Table 3. This duality arises from a tension between the perceived benefits and the risks associated with AI. On one hand, AI’s potential to enhance diagnostic accuracy, improve service efficiency, and advance medical research generates positive emotions, such as awe, appreciation, and optimism for its future development. On the other hand, its perceived instability, operational opacity, and black box nature provoke skepticism and resistance. The inability to comprehend AI’s decision-making processes, coupled with concerns about errors and biases, contributes to an expanding trust gap. This is vividly reflected in public expressions of skepticism regarding the reliability of AI for medical treatment, underscoring the emotional barriers to its integration.

Similarly, the public’s affective responses toward the integration of AI with physicians reflect a complex induterplay of hope and anxiety, which is central to the model’s progression from cognition to emotion. Many view the transformation of traditional clinical workflows through AI collaboration as a positive development, believing it could enable physicians to devote more time to meaningful patient communication, thereby strengthening the physician-patient relationship. However, this optimistic view is counterbalanced by significant concerns. As AI takes on an increasing number of decision-making roles, the public expresses concerns about the potential over-reliance of physicians on AI in clinical decisions, coupled with fears that the technology may eventually replace human healthcare providers. These sentiments reflect deeper anxieties about the loss of professional autonomy and identity, completing a crucial link in the model that connects cognitive perceptions of role boundaries to emotionally charged public discourse.

#### 3.3.3 Behavioral strategies

The affective responses to medical AI ultimately evolve into concrete behavioral strategies, which represent the critical action phase in the public engagement model, completing the feedback loop illustrated in Figure 3. These adaptive strategies, emerging from the interplay between public cognition and emotion, can be categorized into three primary types that facilitate societal negotiation and adaptation.

First, a prevalent strategy that has emerged is cautious acceptance of medical AI. This approach acknowledges the technology’s potential while remaining mindful of its associated risks. This behavoir is reflected in the views of participants who advocate for embracing the benefits of technological convenience, while simultaneously exercising caution and proactively addressing the emerging challenges it presents. With respect to physician-AI collaboration, this translates into a strong consensus in favor of a collaborative augmentation model, where physicians retain leadership in clinical decision-making, leveraging AI as a supportive tool. This model requires physicians to maintain critical thinking, uphold professional autonomy, and carefully evaluate AI-generated recommendations without over-relying on or dismissing them entirely.

Second, the enhancement of AI and information literacy has emerged as a vital coping mechanism. In the era of information overload, both the general public and healthcare professionals face the challenge of filtering and assessing complex AI-related information. The public recognizes the necessity of developing the skills needed to identify credible sources and evaluate their reliability. For healthcare providers, this entails improving their understanding of AI systems, applying critical judgment to AI outputs, and committing to ongoing professional development. As one post highlighted, physicians must cultivate and sustain the critical competency to identify and rectify diagnostic or therapeutic errors made by AI systems. This literacy development is seen as essential for capitalizing on AI’s data-processing strengths while safeguarding independent clinical judgment and preventing undue reliance on technology.

Finally, there is a growing public demand for robust governance and policy frameworks. Heightened awareness of AI’s societal impacts, coupled with varied affective responses, has led to calls for comprehensive regulation. These policy demands center on three critical areas: ensuring decision reliability and transparency, implementing rigorous data security protocols, and clarifying accountability and rights protection mechanisms. Through these advocacy efforts, the public seeks to ensure that medical AI innovations contribute positively to societal well-being, while adhering to principles of fairness, safety, and sustainability.

These three behavioral strategies, cautious acceptance, literacy enhancement, and policy advocacy, form the action component that completes the cyclical process outlined in Figure 3. They not only serve as direct responses to cognitive and affective evaluations but also generate new societal experiences, which in turn feed back into public cognition. This continuous reappraisal and adaptation exemplify the evolving relationship between society and medical AI.

## 4 Discussion and conclusion

### 4.1 A policy-driven public perspective on medical AI

A central finding of this study is the distinctive emphasis placed by the Chinese public on the foundational drivers of medical AI development, such as national strategies, global collaborations, and the digital economy. This focus differs from existing literature, which tends to concentrate on public’ perceptions toward acceptance (26, 35), challenges of implementation (12, 36), and potential applications of AI in medical care (17). In contrast, the Chinese public views AI as intertwined with policy direction, where strategic planning and international collaboration are critical in shaping its trajectory. This aligns with China’s collectivist values and long-term goals, where societal benefits are prioritized over individual concerns (26). This finding is significant, as it highlights the need to understand AI adoption not only through a technological lens but also within the broader socio-political and ethical governance context. It contributes to the existing literature by suggesting that in countries like China, where government policies and national strategies play a dominant role in shaping public perceptions, the AI integration is perceived as a collective and long-term ethical endeavor, rather than merely an isolated technological advancement.

In addition, our findings resonate with existing literature on the public’s concerns regarding the application domains of medical AI. The themes of ‘AI applications in assisted diagnosis and treatment’and ‘AI-driven biomedical research and innovations’align with well-established findings that highlight AI’s potential to enhance diagnostic accuracy (37, 38) and drive scientific progress (20, 39). These insights contribute to the broader discourse on AI in healthcare, consistently emphasizing its transformative potential in improving healthcare delivery and advancing scientific discovery.

Our findings also reveal that the Chinese public recognizes the societal impacts of medical AI, particularly concerns around privacy, risk, and ethics. These concerns resonate with those raised in existing literature, which similarly emphasizes the ethical implications of AI adoption (40). Furthermore, our study offers additional insights into the impact of medical AI on occupational structures and education systems, emphasizing the necessity for medical professionals to adapt their skillsets for effective collaboration with AI. While these findings align with existing discussions (17), they also expand our understanding of the evolving demands on the healthcare workforce and medical education.

### 4.2 Balancing human-centered care and AI’s technical role

This study identifies a defining characteristic of Chinese public perspectives on medical AI: an insistence on safeguarding medicine’s humanistic foundations. While acknowledging AI’s proficiency in technical domains (e.g., diagnostic support, data processing), respondents consistently emphasized its incapacity to replicate quintessentially human attributes—particularly empathy, contextual ethical reasoning, and dignity preservation. Crucially, resistance originates not from technological skepticism, as some studies suggest (1, 13, 41), but from a principled defense of healthcare’s moral core. Consequently, AI is construed strictly as an adjunctive instrument subordinate to physician authority, reflecting recognition of its inherent limitations in moral agency.

Given this foundational concern, the public overwhelmingly favors a collaborative model between physicians and AI wherein physicians retain ultimate authority over medical decision-making. This aligns with established frameworks on human-AI collaboration that emphasize role demarcation (42). Within such models, AI deployment remains ethically permissible only when confined to strictly technical functions (e.g., medical imaging analysis) that neither require nor simulate human relational capacities. Conversely, tasks demanding emotional intelligence or moral discernment are categorically excluded from AI’s operational scope. This functional demarcation constitutes a socioculturally embedded ethical safeguard against technological encroachment on domains requiring irreducible human judgment—particularly within Chinese medical contexts. Consequently, viable medical AI systems must computationally excel in technical domains while consciously preserving physician primacy in relational care, thereby aligning with public expectations to mitigate adoption resistance.

### 4.3 Public behavior strategies in response to medical AI

Our study reveals that the Chinese public’s engagement with medical AI involves not only passive reactions but also proactive moral agency aimed at navigating ethical tensions. This extends beyond literature focusing on emotional reactions (21) and cognitive assessments (15, 22), but rarely explores how these factors transform into proactive behaviors. Three concrete ethical actions: cautious implementation to mitigate algorithmic bias, self-education to combat epistemic injustice, and policy advocacy demanding institutional accountability. These behaviors reflect a deeper level of ethical agency than previously acknowledged, moving beyond passive acceptance of AI in healthcare.

This ethical agency manifests through the CAB framework’s ethical feedback loop: Public recognition of medical AI’s moral limitations (e.g., limited affective engagement or decision opacity) generates concerns, which mobilize self-protective strategies that recursively reshape cognitive frameworks. Of particular significance is how these adaptive behaviors create a self-reinforcing cycle wherein public engagement continuously redefines societal perceptions of AI’s role in health systems. Most notably, the prominence of policy advocacy exemplifies China’s distinctive socialist governance frameworks. In this system, state-society synergy effectively translates public ethical demands into formal regulatory mechanisms, demonstrating a unique pathway for institutionalizing moral agency in digital health governance.

Building on these insights, we argue that China’s governance frameworks illustrate how bottom-up ethical agency can be integrated into top-down regulatory mechanisms. This state-society co-regulation model provides a replicable template for translating civic moral intuitions into actionable governance protocols, such as converting policy advocacy into mandatory algorithmic audits.

### 4.4 Limitations and future directions

While this study provides valuable insights into the public’s concerns regarding medical AI, its emotional characteristics, and the CAB mechanisms driving engagement, several limitations must be acknowledged.

#### 4.4.1 Sample diversity, coverage and temporal bias

The generalizability of our findings is constrained by the demographic bias inherent in Sina Weibo data. While Sina Weibo predominantly captures digitally privileged demographics, particularly urban, highly educated individuals, it underrepresents digitally marginalized groups, such as older adults, people with lower levels of education, and rural residents. For instance, Weibo user express positive views about AI’s potential to enhance healthcare, underrepresented groups may hold more negative perceptions, focusing on fundamental issues such as basic healthcare access, the digital divide, and the affordability of AI-driven services.

Furthermore, in terms of human-AI collaboration, marginalized groups are likely to place greater value on doctor-led diagnoses, seeing them as a core aspect of physician responsibility and patient care. The involvement of AI may thus be perceived as a reduction in the physician’s duty, potentially deepening distrust in the technology. As a result, these groups are more likely to adopt passive or defensive strategies, such as avoiding AI services or relying on intermediaries (e.g., family members or community workers) due to mistrust, rather than engaging in proactive behaviors like cultivating technological rationality and cautious awareness.

To address this limitation, future research could adopt a mixed-methods approach, combining the qualitative insights of grounded theory with the broader statistical coverage of nationally representative surveys. Incorporating stratified sampling and conducting offline interviews in healthcare settings could offer a more nuanced understanding of public perceptions across various demographic groups, including urban versus rural populations, age, and education levels. This would provide a more comprehensive and representative portrayal of public attitudes toward medical AI. In addition to addressing demographic bias, the dynamic and event-driven nature of social media means our analysis cannot capture temporal shifts in discourse and sentiment, which may further influence public perceptions of medical AI. To overcome this limitation, future research should integrate diverse data sources, such as longitudinal surveys, to track the evolution of public sentiment and discourse over time while also ensuring more comprehensive representation of different demographic groups.

#### 4.4.2 Role of cultural context in shaping public engagement

The role of cultural context in shaping public engagement with medical AI warrants further investigation within our theoretical framework. The policy-driven perspective identified in this study is closely tied to the specific characteristics of China’s healthcare system, where the government plays a central role in shaping public perceptions of medical AI. As a result, public coping strategies prominently reflect advocacy for government-led policy initiatives. Additionally, the Confucian value of benevolence, which emphasizes physician compassion and ethical responsibility, provides a distinct humanistic benchmark for evaluating AI’s role in healthcare.

We hypothesize that the prominence of different components within our model will vary significantly across cultural contexts. In individualistic societies with market-driven healthcare systems (e.g., the United States), cognitive appraisals of AI are likely to emphasize concerns about algorithmic transparency, while affective responses are expected to focus on issues of personal data autonomy. This would likely result in behavioral strategies centered around demands for corporate accountability. In contrast, in societies with social market economies (e.g., Germany), the cognitive foundation may prioritize equity and fairness, with affective responses more closely linked to social solidarity. Consequently, public behavior may focus on ensuring robust data protection to serve the collective good.

Future research should include cross-cultural comparisons across diverse countries (e.g., Germany, India, the United States) to test these hypotheses. Rather than merely documenting attitudinal differences, the aim should be to examine how cultural orientations specifically influence the cognitive, affective, and behavioral pathways outlined in our model. This mechanism-focused approach is essential for developing governance frameworks for medical AI that are both culturally sensitive and globally applicable.

## Data Availability

The raw Weibo data supporting the findings of this study are not publicly available due to the terms of service of its API. However, the data are available from the TJ (jting1681@163.com) upon reasonable request.
